# Variability of marine heatwaves’ characteristics and assessment of their potential drivers in the Baltic Sea over the last 42 years

**DOI:** 10.1038/s41598-024-74173-2

**Published:** 2024-09-28

**Authors:** Behzad Bashiri, Amirhossein Barzandeh, Aarne Männik, Urmas Raudsepp

**Affiliations:** Department of Marine Systems, Tallinn University of Technology, Tallinn, 12618 Estonia

**Keywords:** MHW, Baltic Sea, Climate oscillations, Heat flux, Ocean sciences, Physical oceanography

## Abstract

This study examined Baltic Sea Marine Heatwaves (MHWs) using 42 years of satellite-derived sea surface temperature (SST) data. We found that MHWs in warmer months are more intense but shorter compared to MHWs in cooler months. Also, MHWs predominantly affect offshore areas in warmer months, whereas MHWs predominantly impacting coastal seas in cooler months, especially along the eastern coast. Our analysis of interannual variability revealed that, unlike in many other basins worldwide, Baltic MHWs tend to maintain a constant intensity, while their spatial extent has significantly increased over the last few decades. Shortwave radiation notably influences MHW intensity and spatial extent, with additional impacts from longwave radiation in cooler months and latent heat flux in warmer months. Northern Hemisphere teleconnections exhibit stronger correlations with MHWs in the Baltic Sea compared to global-scale climate oscillations, with the Eastern Atlantic pattern having a particularly significant effect on MHW variability in the region.

## Introduction

A MHW refers to a prolonged period of unusually warm ocean temperatures within a specific region, often lasting days to months, with temperatures significantly above the average seasonal conditions^1^. MHW, like heatwaves on land, can cause big problems for the oceans. They can seriously affect sea animals and plants, the variety of life in the ocean, the usual weather in an area, and human life in the coastal area^2–6^. On the other hand, MHW holds immense significance in both understanding climate change and studying oceanography. These events serve as critical indicators of the changing climate’s impact on the world’s basins. By examining the frequency, duration, and intensity of these heatwaves, valuable insights can be gained into how rising global temperatures affect the delicate balance of our basins^7–9^. These events provide essential data for oceanographers studying the complexities of ocean dynamics, including circulation patterns, heat transfer mechanisms, and the interplay between the atmosphere and the sea^10^.

**Fig. 1:**
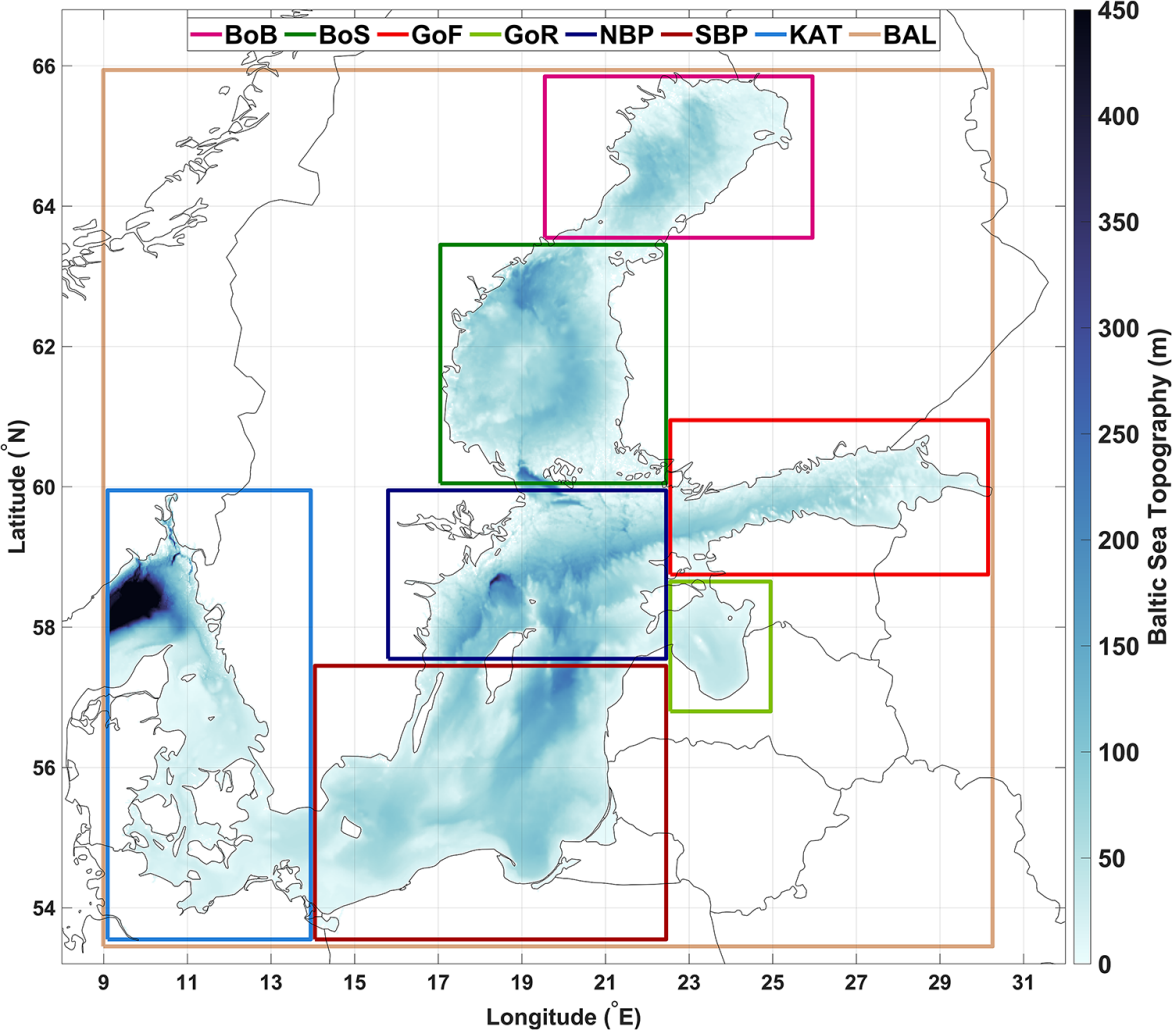
Map of the study area, the Baltic Sea, includes subbasin: the Bothnian Bay (BoB), the Bothnian Sea (BoS), the Gulf of Finland (GoF), the Gulf of Riga (GoR), the Northern Baltic Proper (NBP), the Southern Baltic Proper (SBP), the Kattegat basin (KAT), and the entire Baltic Sea (BAL). The colour scale shows the depth according to the General Bathymetric Chart of the Oceans (GEBCO)^45^ and the final map were generated using MATLAB r2022b programming platform^46^.

As climate change intensifies^11^, comprehending the behavior and effects of MHWs becomes increasingly essential in predicting future oceanic trends and their broader implications for the planet. However it is important to note that climate change, as a global phenomenon, reveals itself differently in various regional basins around the world due to the complex interactions between the atmosphere, oceans, land, and local geographical features^12–14^. Therefore, it is crucial to consider the effects of MHWs within regional basins^15,16^. The initial detection of MHWs in a specific basin offers a detailed understanding of its climatic and oceanographic conditions. This typically involves analyzing historical SST records using a type of extreme value analysis to identify periods where temperatures exceed a predefined percentile threshold—commonly set at the 90th percentile—for at least five consecutive days, defining a prolonged warm water event^17,18^. Recent studies, such as^19–23^, examining MHW characteristics within different regional basins, often use this 90th percentile threshold. However, raising the threshold to the 95th or 99th percentile can isolate more extreme MHW events for more specific purposes^24–26^. Regardless of the chosen percentile or minimum consecutive days used for detecting MHWs in a basin, it is crucial to establish a climatology baseline for defining thresholds. Typically, there are two options for setting this baseline: fixed and moving. Using a fixed baseline is more advantageous for studying long-term MHW characteristics, as it reflects the inherent long-term trends in SST data during the MHW detection process and provides a more appropriate basis for comparing MHW characteristics across different time intervals, particularly in light of climate change. However, only with a moving baseline can we consistently define marine heatwaves as rare extreme events^27^. In addition, selecting a fixed baseline period can be challenging due to the bias introduced by interannual variations in SST, which are influenced by oceanic and climatic phenomena that do not occur in the same way every year or decade^28,29^.The Baltic Sea, which includes 7 subbasins (Fig. 1) and is located in Northern Europe, stands as one of the largest brackish water bodies globally. It is characterized by its unique geographical and climatic conditions and experiences notable temperature and heat variability across its diverse regions^30,31^. The Baltic Sea’s complex interplay among freshwater input, saline waters, and distinct circulation patterns provides an intriguing opportunity to explore regional climate fluctuations and their impact on ocean dynamics. This aspect positions the Baltic Sea as a pivotal subject within conversations addressing environmental changes and initiatives aimed at fostering sustainability^32–36^.

The spatial distribution of extreme marine heat events has expanded significantly over the past century, with many regions, including the Baltic Sea, experiencing extreme heat levels that were once rare^37^. Some recent studies have reported that a systematic study is still needed to achieve a comprehensive understanding of marine heatwaves in the Baltic Sea^38,39^. This comprehensive understanding is crucial for providing a proper basis for further work, such as predicting the probability of future warming events and the effect of climate change in the Baltic region. This could explain why, recently, the rate of publication of studies related to MHWs in the Baltic Sea has increased significantly. Gröger et al. (2024)^40^ using hindcast data for the period 1980–2016, indicated the seasonal variability of MHWs in the Baltic Sea and examined the drivers, finding that summer MHWs are primarily driven by local meteorological conditions and winter MHWs by warm, moist air advection from the North Atlantic. Travkin et al. (2024)^41^ analyzed MHWs based on regional reanalysis data of the Baltic Sea from 1993 to 2022. They found out that there is a weak correlation between the annual mean intensity and the total duration of MHW. In areas with a large number of MHWs, their duration is relatively short. However, in areas where there are a small number of MHWs, their mean duration significantly increases. Dabulevičienė and Servaitė (2024)^42^ examined MHWs in the southeastern Baltic Sea from 1993 to 2023, utilizing in situ data and satellite observations. Their study revealed an increase in both the frequency and intensity of MHWs in recent years, with recent decades showing longer and more severe heatwaves, impacting both coastal and open waters. Pinto et al. (2024)^43^ investigated MHWs in the Western Baltic Sea from 1950 to 2022 using a 3D-hydrodynamic model. They found that MHWs have become more frequent and longer-lasting, occurring in all seasons, and that some extreme events combined with hypoxic conditions, pose significant ecological risks. They also noted that the most significant MHW in terms of intensity and duration occurred in early 2020. Additionally, they highlighted variability in spatial trends, indicating that surface MHWs were more frequent in the southern parts of the Western Baltic Sea, near the German coast, while longer-duration and higher-intensity events were more prevalent around southern and southwestern Sweden. Safonova et al. (2024)^44^ investigated the impact of increasing MHWs on the Baltic Sea seabed using reanalysis data in recent decades, revealing that there is a statistically significant correlation between cases with low oxygen content in the coastal zones of the Baltic Sea and MHW events.

In addition, the significance of the MHW in the Baltic Sea from different perspectives can be traced sporadically in previous research. For example, Humborg et al. (2019)^47^ illustrated that the MHW of 2018 in the Baltic Sea triggered CO2 and CH4 fluxes in the coastal zones that were comparable with maximum emission rates found in other hot spots, such as boreal and arctic lakes and wetlands. In addition, Suursaar (2020)^48^ stated that the occurrence of coastal upwellings in the Gulf of Finland caused a local cooling effect during the simultaneous presence of a MHW, occasionally mitigating its overheating effects both in the sea and at the marine-land boundary. However, the co-occurrence of MHWs and coastal upwellings in the eastern Baltic Sea increases heat transfer from the atmosphere to the water mass, and rising MHW extremes and coastal upwellings may stress ecosystems. Cahill et al. (2024) examined the interplay between MHWs and phytoplankton blooms in the Arkona Sea during 2018, using satellite data and modeling^49^. Their study found that the first MHW in May created optimal conditions for phytoplankton growth, which was sustained by nutrient transport processes, while subsurface temperature anomalies from MHWs influenced the bloom’s duration and intensity. These kinds of incidents motivate researchers to conduct more extensive Baltic Sea MHW studies. In this regard, this study aims to detect and analyze Baltic Sea MHWs using long-term satellite datasets to achieve a comprehensive understanding of these events. Furthermore, this study aims to explore relationships between MHW variability and several potential drivers to enhance insight into their origins and their inherent correspondence with climate patterns. This study addresses the long-term variability of MHW characteristics in the Baltic Sea, which have significant implications for marine ecosystems, fisheries, and coastal communities. By utilizing long-term satellite datasets, we can capture a more accurate and detailed picture of MHW events, allowing us to identify patterns that may not be evident in previous studies. Additionally, understanding the drivers behind MHW variability will enable us to predict future events more reliably, providing essential information for mitigating their impacts and adapting to the changing climate. The insights gained from this research could also contribute to the broader scientific community’s understanding of how marine heatwaves are linked to global climate dynamics, ultimately supporting more informed policy decisions and conservation strategies in the Baltic Sea region.

## Results

Four key characteristics of MHWs in the Baltic Sea were extracted based on the detected records from satellite data (1982–2023). The criteria for evaluating MHWs during this long-term study involved identifying events that exceeded the 90th percentile of SST for at least 5 consecutive days. The baseline period for defining the 90th percentile, set from 1992 to 2002, was chosen based on specific criteria, with the rationale thoroughly detailed in the Data and Method section of the present study.

### Seasonal variability of MHW

**Fig. 2:**
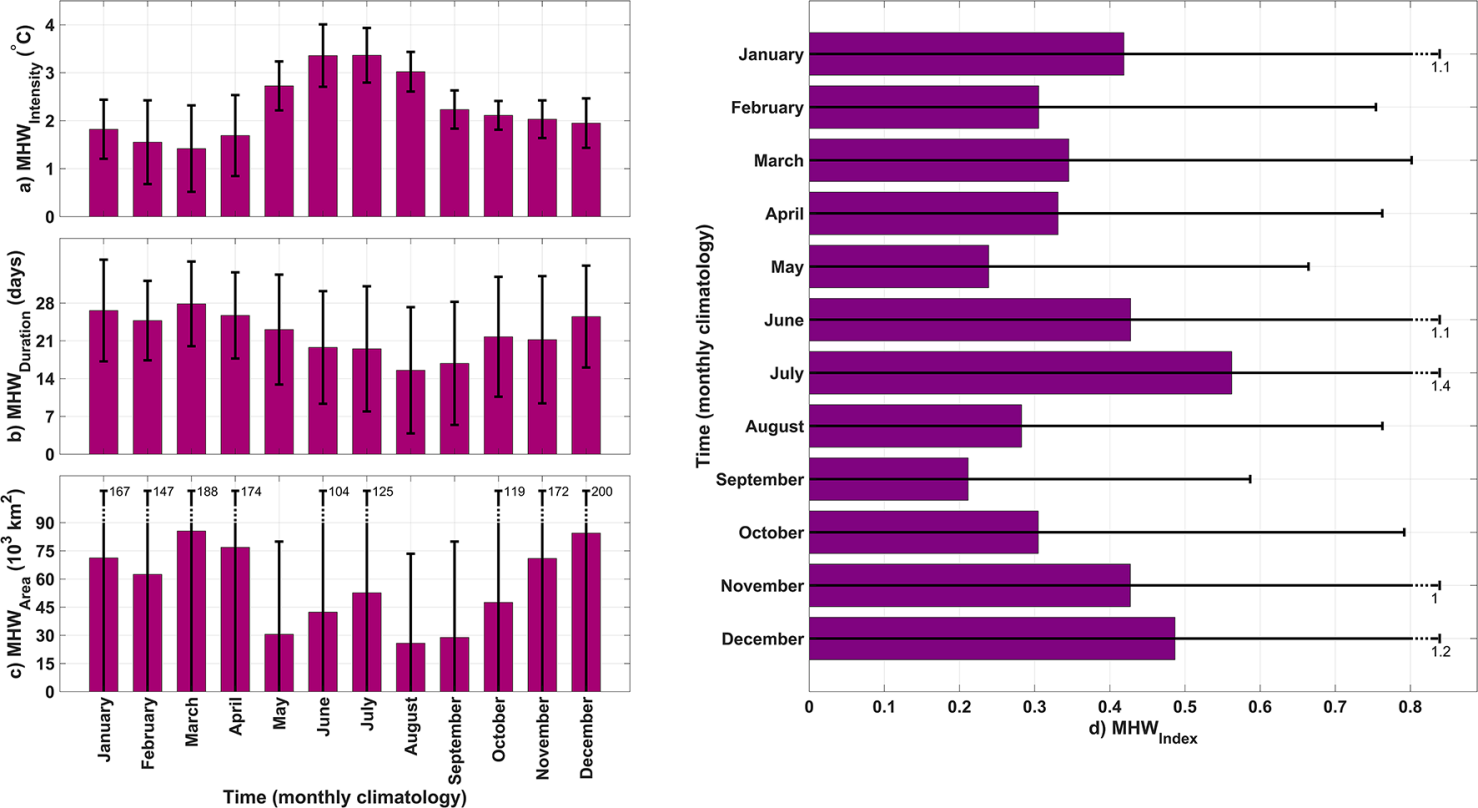
Monthly averaged variations of MHW characteristics: (**a**) MHW_intensity_, (**b**) MHW_duration_, (**c**) MHW_area_, and (**d**) MHW_index_. The black lines represent the standard deviation.

The intensity of MHWs exhibits a pronounced seasonal cycle, with a peak occurring during the summer months, particularly in June and July. The mean intensity of detected MHWs in the Baltic Sea consistently exceeds 3 °C across all identified times and spaces. Conversely, the minimum MHW_intensity_ occurs in March, with a mean intensity of ~ 1.5 °C (Fig. 2a). MHW_duration_ also has a smoother seasonal pattern, peaking in March and dipping in August (Fig. 2b). One-month shift MHW_intensity_ and duration seasonality tendencies are inverse. MHW_area_ peaks in cooler months like March, April, and December and decreases in August and September (Fig. 2c). The monthly averaged annual cycle of the MHW index during the study period shows peaks in both June and July, as well as in December and January, highlighting the significant occurrence of MHWs in both summer and winter over the last four decades (Fig. 2d).

**Fig. 3:**
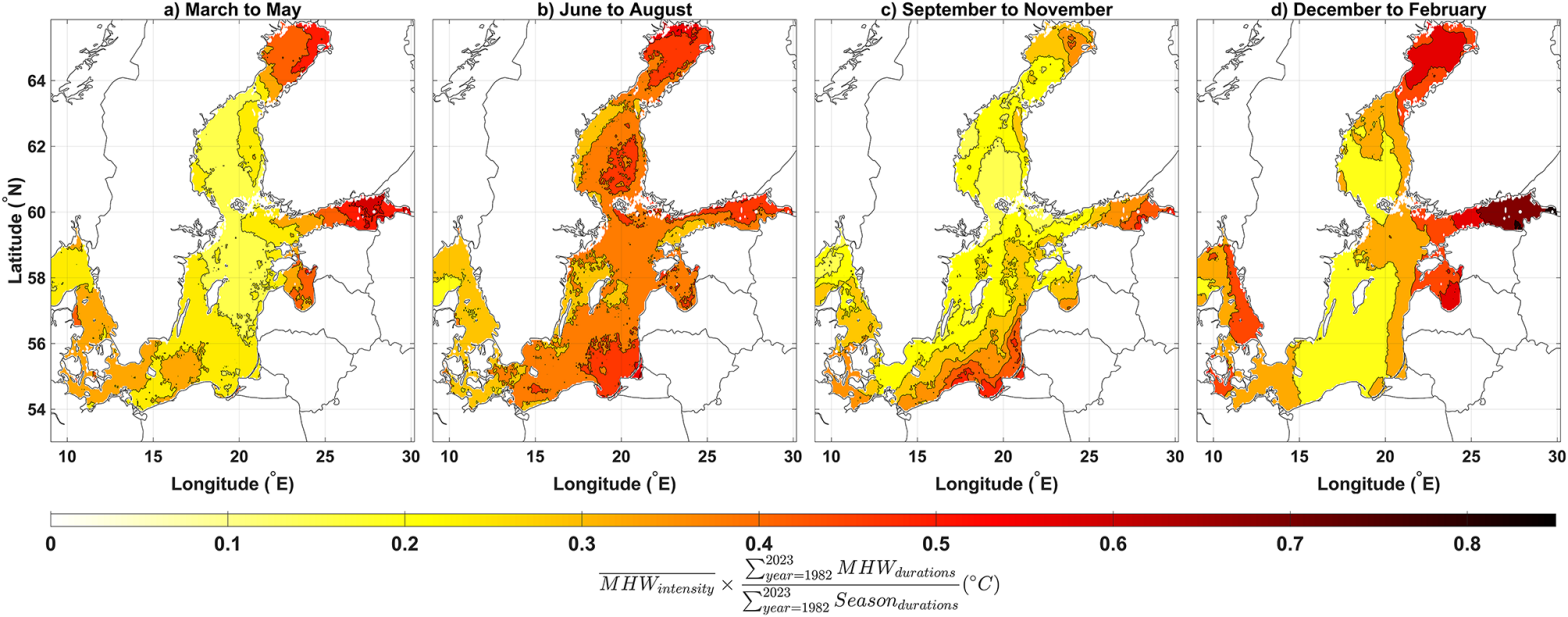
Spatial distribution of the seasonal MHW patterns: (a) March-May, (b) June-August, (c) September-November, and (d) December-February.

To better assess seasonal MHW variability in the Baltic Sea and visualize MHW spatial distribution, we eliminated the area-related element from our MHW_index_ definition. For each of the four seasons, we created mean MHW_intensity_ maps weighted by MHW_duration_ relativity (Fig. 3). Results reveal that MHW spatial distribution varies with season as well. BoB, GoF, KAT, and SBP usually see more MHWs in the spring. In contrast, central Baltic Sea MHW_intensity_ are moderate (Fig. 3a). Summer MHW_intensity_ is higher, with western boundaries experiencing less than eastern boundaries (Fig. 3b). The central Baltic’s mean MHW_intensity_ decreases significantly in the fall (Fig. 3c). BoB, GoF, and KAT have heightened MHW in winter, similar to spring patterns with intensified values (Fig. 3d).

### Interannual variability of MHW

**Fig. 4:**
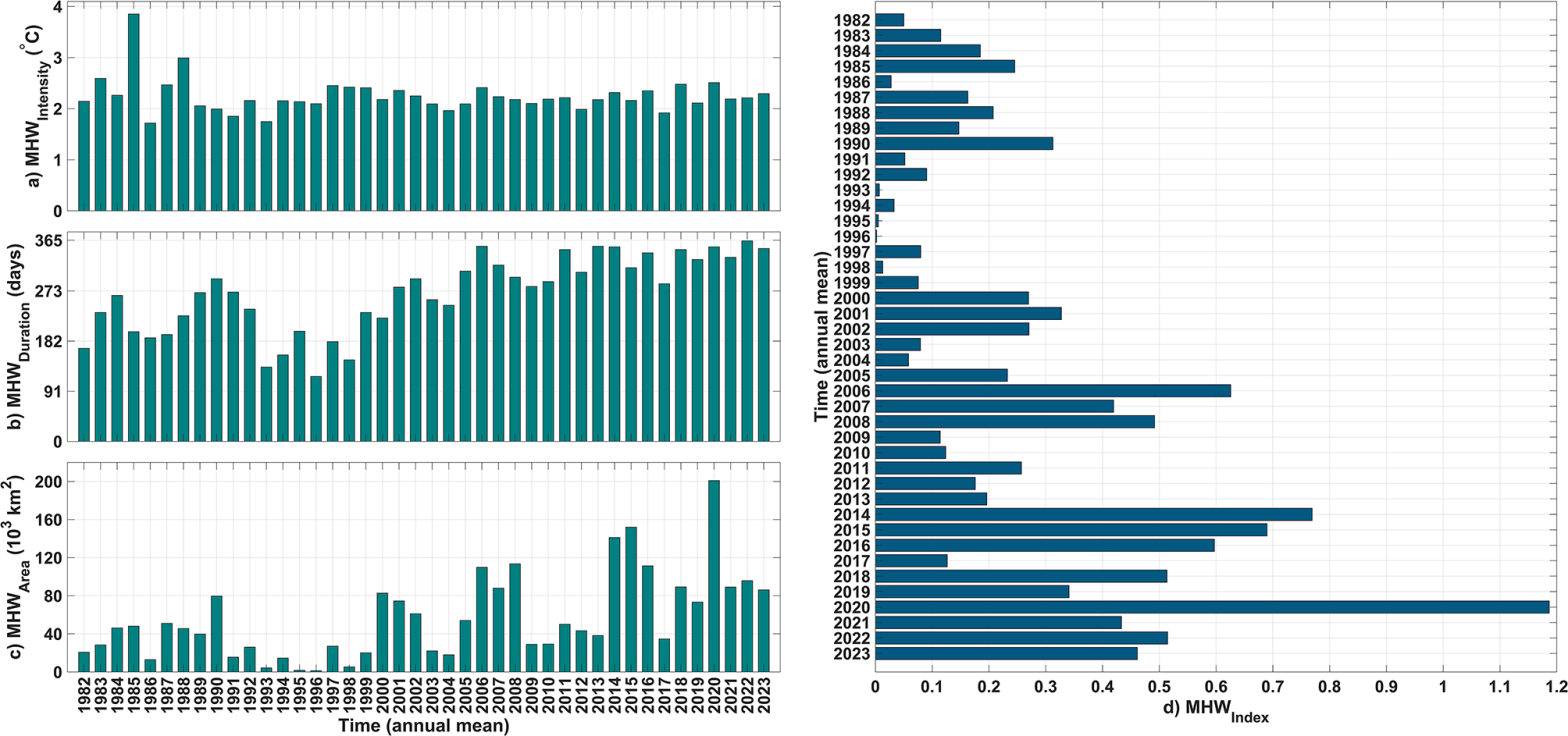
Interannual variations of MHW characteristics: (**a**) MHW_intensity_, (**b**) MHW_duration_, (**c**) MHW_area_, and (**d**) MHW_index_.

Particularly high MHW_intensity_ was observed in 1985 and 1988 (Fig. 4a). Only intensities show modest changes, although the mean interannual variability of MHW_intensity_ over the last 3 decades is not very high. Interannual variance in detected MHW_intensity_ is moderate. The interannual cycles of detected MHW_duration_ vary from 90 days in 1996 to nearly whole-day incidents in 2022 (Fig. 4b). These values may not necessarily indicate significant durations because our analyses average all detected MHW grid points, not only significant ones. That means even if only one grid-point in the Baltic Sea detects an MHW on a given day, the day is included in the cumulative MHW_duration_ computation. In contrast, interannual MHW_area_ variability fluctuates more. Due to the Baltic Sea’s vast area, MHWs can be discovered in many places, but not all are important (Fig. 4c). In 2020, the Baltic Sea had the most MHW coverage in 40 years. A considerable, patchy MHW trend seems sensible. The Baltic Sea MHW_index_ fluctuates year-to-year, typically patchily every four years, with a growing trend. In the last 4 decades, the most significant MHW events were in 2020 (Fig. 4d).

### Correspondence of Baltic MHWs with Heat Flux components

**Fig. 5:**
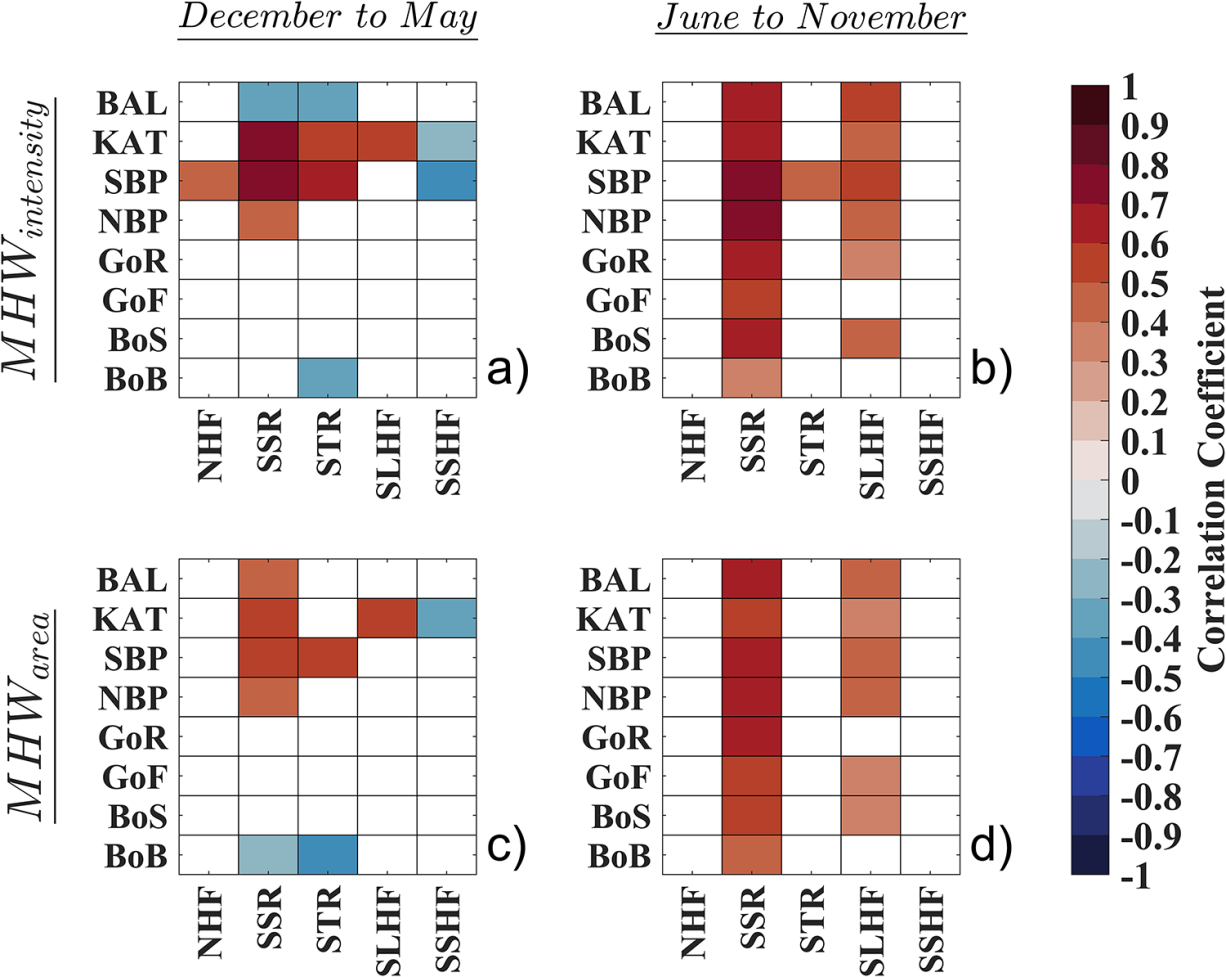
Correlations between sea surface heat flux components and the MHW characteristics variabilities: (**a**) sea surface heat flux component and MHW_intensity_ in cool months (December to May), (**b**) sea surface heat flux component and MHW_intensity_ in warm months (June to November), (**c**) sea surface heat flux component and MHW_area_ in cool months, (**d**) sea surface heat flux component and MHW_area_ in warm months.

The relationship between the time series of spatially averaged sea surface heat flux components—including surface short-wave solar radiation (SSR), surface long-wave thermal radiation (STR), surface latent heat flux (SLHF), surface sensible heat flux (SSHF), and surface net heat flux (NHF)—and MHW characteristics was examined to assess the impacts of internal drivers on MHW variability in the Baltic Sea, both for cool and warm months separately. Initial results indicated that the correlation coefficients between any of the sea surface heat flux components and MHW_duration_ and MHW_index_ were not significant in any of the subbasins, with P-values exceeding 0.05. Consequently, these correlations were excluded from the results presented in this study (Fig. 5).

However, SSR exhibited a considerable correlation (*r* = 0.7–0.8) with MHW_intensity_ during the cool months in the southern subbasins, including KAT and SBP. Additionally, STR showed a slightly lower correlation (*r* = 0.5–0.7) with MHW_intensity_ in the same subbasins and season (Fig. 5a). In contrast, during the warm months, the highest correlation (*r* = 0.7–0.8) between MHW_intensity_ and SSR is observed in the SBP and NBP subbasins, while SSR remains less correlated with all other subbasins. Additionally, SLHF shows a notable correlation with MHW_intensity_, though it is weaker compared to SSR, across the Baltic Sea and its subbasins (Fig. 5b). For MHW_area_, the correlation patterns are similar to those observed for MHW_intensity_, with generally lower correlation values (maximum of *r* = 0.5–0.6) with the corresponding sea surface heat flux components, in both cool and warm months (Fig. 5c & d). Other sea surface heat flux components do not show significant correlations with MHW characteristics.

### Signature of External drivers in the Baltic MHWs variability

**Fig. 6:**
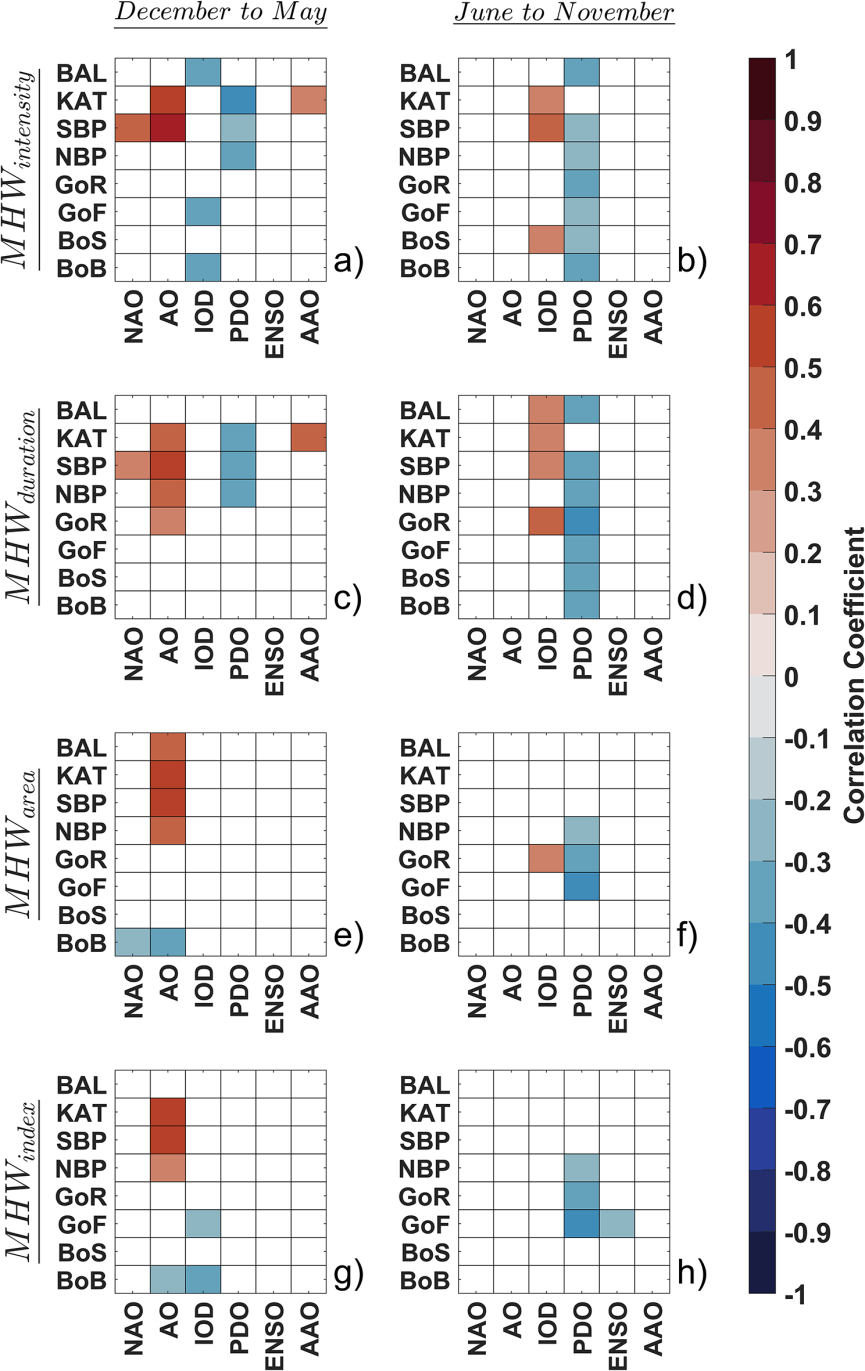
Correlations between the variability of the global-scale climate oscillations and the MHW characteristics: (**a**) the global-scale climate oscillations and MHW_intensity_ in cool months (December to May), (**b**) the global-scale climate oscillations and MHW_intensity_ in warm months (June to November), (**c**) the global-scale climate oscillations and MHW_duration_ in cool months, (**d**) the global-scale climate oscillations and MHW_duration_ in warm months, (**e**) the global-scale climate oscillations and MHW_area_ in cool months, (**f**) the global-scale climate oscillations and MHW_area_ in warm months, (**g**) the global-scale climate oscillations and MHW_index_ in cool months, (**h**) the global-scale climate oscillations and MHW_index_ in warm months.

We analyzed the relationships between global-scale climate oscillations and the variability of MHW characteristics in the Baltic Sea. These climate oscillations investigated in this study include the North Atlantic Oscillation (NAO), Arctic Oscillation (AO), Indian Ocean Dipole (IOD), Pacific Decadal Oscillation (PDO), El Niño-Southern Oscillation (ENSO), and Antarctic Oscillation (AAO). Figure 6 illustrates the correlation analysis between MHW characteristics and these global-scale climate oscillations, with separate evaluations for cool and warm months. The results highlight a distinct seasonal variation in how these climatic oscillations influence MHWs. During the cool months, MHW_intensity_ shows a significant correlation with AO in KAT and SBP, as well as with NAO in SBP, with correlation coefficients ranging from 0.5 to 0.7 (Fig. 6a). Conversely, in the warm months, MHW_intensity_ does not exhibit a significant correlation with either AO or NAO. However, a slight positive correlation (*r* = 0.3–0.5) is observed with IOD in KAT, SBP, and BoS (Fig. 6b). Additionally, a slight negative correlation between MHW_intensity_ and PDO is calculated in some sub-basins across both cool and warm months. These correlation patterns, though varying slightly across neighboring sub-basins, are also evident in the analysis of MHW_duration_ (Fig. 6c and d). During the cool months, MHW_area_ exhibits a positive correlation with AO in the southern sub-basins (KAT, SBP, NBP) and BAL, while a small negative correlation is noted between MHW_area_ and both AO and NAO (Fig. 6e). In contrast, during the warm months, the MHW area generally does not display significant correlations with the large-scale climatic oscillations, except for a low positive correlation with IOD in GoR and a slight negative correlation with PDO in the central and eastern sub-basins (NBP, GoR, and GoF) (Fig. 6f). The MHW_index_ during the cool months shows a positive correlation with AO in KAT and SBP, with a weaker correlation extending to NBP (Fig. 6g). In the warm months, the MHW_index_ only shows a slight negative correlation with PDO in the central and eastern sub-basins (Fig. 6h). Figure 6 also underscores that the correlation between MHW characteristics in the Baltic Sea and both the AAO and ENSO is almost consistently insufficient and negligible.

**Fig. 7:**
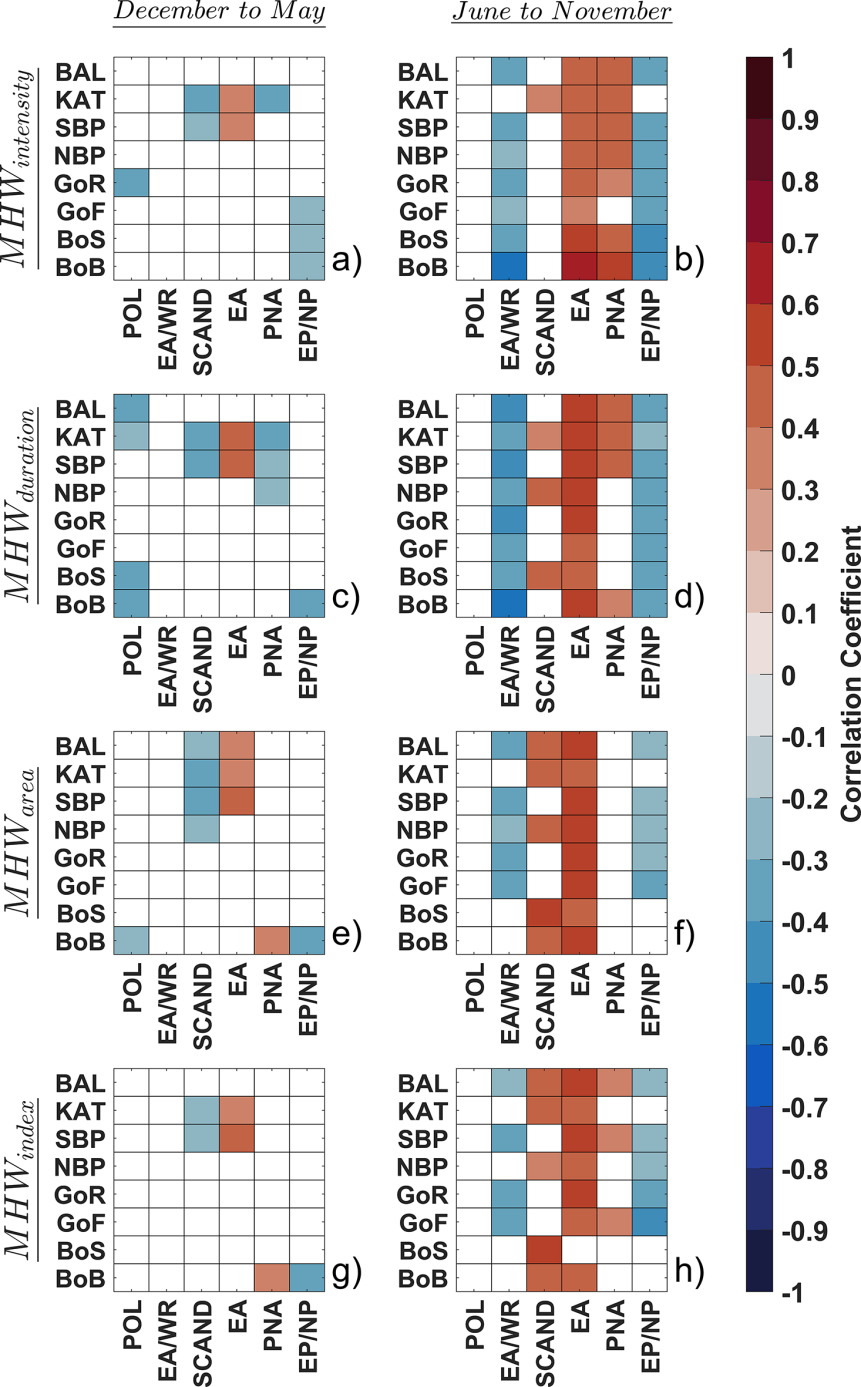
Correlations between the variability of the northern hemisphere teleconnection patterns and the MHW characteristics: (**a**) the northern hemisphere teleconnection patterns and MHW_intensity_ in cool months (December to May), (**b**) the northern hemisphere teleconnection patterns and MHW_intensity_ in warm months (June to November), (**c**) the northern hemisphere teleconnection patterns and MHW_duration_ in cool months, (**d**) the northern hemisphere teleconnection patterns and MHW_duration_ in warm months, (**e**) the northern hemisphere teleconnection patterns and MHW_area_ in cool months, (**f**) the northern hemisphere teleconnection patterns and MHW_area_ in warm months, (**g**) the northern hemisphere teleconnection patterns and MHW_index_ in cool months, (**h**) the northern hemisphere teleconnection patterns and MHW_index_ in warm months.

Additionally, we analyzed the relationships between northern hemisphere climate teleconnections, including Polar/Eurasia (POL), East Atlantic/Western Russia (EA/WR), Scandinavia (SCAND), East Atlantic (EA), Pacific/North American (PNA), and East Pacific-North Pacific (EP/NP) patterns. Figure 7 illustrates the correlation analysis between MHW characteristics and northern hemisphere climate teleconnections, with separate evaluations for cool and warm months.

During the cool months, MHW characteristics generally exhibit low correlations with these teleconnections, except for a small positive correlation (*r* = 0.4–0.6) between MHW_intensity_ and MHW_duration_ with EA in KAT and SBP. These positive correlations extend to MHW_area_ and MHW_index_ in the same sub-basins, though with lower correlation coefficients. Additionally, there are small negative correlations between MHW_duration_ and POL in GoR, as well as with SCAND in KAT and SBP. Similarly, negative correlations are observed between MHW_duration_ and PNA in KAT, and between EP/NP and MHW_duration_ in GoF, BOS, and BoB. Overall, during the cooler months, the number of sub-basins showing even small correlations between northern hemisphere teleconnections and MHW characteristics is limited (Fig. 7a, c, e and g).

In contrast, during the warm months, the correlation coefficients are generally higher. The results indicate a positive correlation between MHW_intensity_ and EA and PNA in the Baltic Sea, with stronger correlations in BoB that gradually decrease toward the southern sub-basins. Conversely, the correlation with EA/WR and EP/NP is significantly negative in the Baltic Sea sub-basins, with the strength of these correlations also decreasing from the northern to the southern sub-basins (Fig. 7b). These correlative patterns are largely consistent during the warm months, with some shifts in neighboring sub-basins and a significant increase in correlations between SCAND and both MHW_duration_ and MHW_area_ (Fig. 7d and f). MHW_index_ shows a strong positive correlation with EA across all sub-basins, though slightly weaker in BOS and KAT. Additionally, SCAND exhibits a small positive correlation with the MHW_index_ in BoS and KAT, with weaker correlations calculated in BoB and NBP (Fig. 7h).

## Discussion

The detection of MHWs primarily relies on SST data^17^. To identify MHWs from SST data, researchers typically define baseline temperature conditions and determine thresholds for MHW events. Generally, there are two options for defining the baseline temperature in MHW detection: time-fixed baseline and time-moving baseline^26,50^. A time-fixed baseline refers to a static, historical reference period used to define climatological norms. Typically, this baseline period spans several years, such as 10 to 30 years, during which the mean and variability of SST are calculated. A fixed baseline provides a stable reference point, facilitating comparisons across different studies and regions. In contrast, a dynamic baseline, also known as a shifting or rolling baseline, continuously adjusts the reference period to account for recent SST changes. However, frequent updating of the baseline can make it challenging to compare MHW characteristics over long timescales within a region^18^. This means that the detected results for MHW events may not provide comparable characteristics for further analysis. Considering the initial aim of the present study, which was to provide a comprehensive and long-term overview of MHWs and the variability of their characteristics on different temporal and spatial scales in the Baltic Sea, an improved fixed baseline was defined to reduce the potential bias of climatic variation in the detection procedure. In this regard, an initial review of the literature indicated that the temperature variation of the Baltic Sea is closely related to two climate oscillations: the Atlantic Multi-decadal Oscillation (AMO) and NAO^38^. Therefore, in the pre-processing phase of this study, these two oscillations were evaluated. It was found that, to define a fixed baseline lasting at least 10 years, the optimum period for minimizing the combined influence of the AMO and NAO indices is an 11-year period starting from 1992.

The study found seasonal patterns with varying peaks of MHW characteristics: summer heatwaves are more intense but shorter, while winter heatwaves are weaker but longer. In this regard, considering the MHW_area_ as a third characteristic is crucial. The Baltic Sea experiences the most MHW events between December and March, with fewer events in the summer and spring. This finding is consistent with a recent study that analyzed MHWs in the Baltic Sea using Baltic Reanalysis data based on the NEMO model^41^. Our study further confirms the significance of MHWs in the colder months of the Baltic Sea based on the satellite data. Nevertheless, MHW events in the Baltic Sea are better understood with the MHW_index_ defined in the present study. The MHW_index_ variabilities show that MHWs in the Baltic Sea peak in June and July, and also January and December, according to the monthly mean climatology of the MHW_index_. This indicates that while the high intensity of MHWs is more pronounced in summers, winters contain longer durations and larger areas of MHWs in the Baltic Sea. Previously, Gröger et al. (2024) found that summer and winter MHWs in the Baltic Sea are driven by different factors, which can lead to distinct characteristics in each^40^. The present study also illustrated that the spatial variability of MHWs in cooler months is different from that in warmer months. As illustrated by the spatial distribution of seasonal MHW patterns, summer MHWs mainly influence SST in offshore areas which is consistent with the findings of Gröger et al. (2024)^40^. In addition, in the southeastern part of the Baltic Sea, the more pronounced MHWs during the warm months align with findings from a very recent study that used in situ data to highlight the significance of MHWs in that area^42^. The seasonally separated spatial patterns of MHWs in this study (Fig. 3) indicate that MHWs are more pronounced in BoB, GoF, and GoR compared to BoS, NBP, and SBP during the cool months. Additionally, during these months, the coastal areas of BoS, NBP, and SBP are more affected by MHWs than their open-sea areas. However, in the summer (June-August), MHWs are more significant in the open-sea areas of each subbasin, with some, like BoS, showing greater significance offshore compared to coastal regions. This suggests that, in general, coastal waters are more sensitive to MHWs during cool months, while offshore areas are more affected during warm months.Our results show, MHW_intensity_ in the Baltic Sea does not vary significantly between years, except for unusual years like 1985 in the early part of the research period. MHW_area_ and MHW_duration_ are usually less in early years with peak intensity, such 1985. Recent years have seen more relative peaks in the variation of the Baltic Sea MHW characteristics. After 2000, MHW_duration_ peaked in 2006, 2014–2015, 2018, 2020, and 2022. Considering the MHW_area_ to improve our interpretation, the Baltic Sea reveals a definite increasing manner. The entire Baltic Sea covers around 400,000 km², and the peak in MHW_area_ occurred in 2020, with an average coverage of nearly 200,000 km². Since at least one data grid point in the Baltic Sea experienced MHW for at least 365 days in 2020, 50% of the sea area experienced it almost every day. Based on interannual variability, the average intensity of MHW-covered areas in 2020 exceeded the 90th percentile threshold of the usual SST by 2.5 degrees. These findings reveal that 2020 was the year with the most significant Baltic Sea MHWs from 1982 to 2023. Additionally, in the past two decades, the MHW_index_ has increased in a patchy manner, with elevated values occurring at intervals of every 3–5 years and there is a relative peak in each patch with MHW_index_ values much higher than in the preceding years.

Overall, the results indicate that the mean MHW_intensity_ has remained stable over the past four decades, while the area covered by MHWs in the Baltic Sea has increased. This finding is not entirely unexpected, as it aligns with observations from other regions where MHW trends have shown variability. Interestingly, a recently published paper reported a decreasing trend in MHW_intensity_ in the marginal seas of China^51^. In addition, studies in other regions have shown an increasing trend, but this trend was attributed to other MHW characteristics, such as frequency and duration, rather than to significant changes in MHW_intensity_^19,20,52^. These variations underscore the complex and region-specific nature of MHW behavior, making it clear that stable or even decreasing MHW_intensity_ is not necessarily anomalous. While most criteria for detecting MHWs in the present study were consistent with previous research, the key difference lies in the selection of the baseline period. Unlike typical approaches that rely on a standard number of decades, this study aimed to identify a more appropriate baseline period, thereby increasing confidence in the findings. This new insight underscores the importance of choosing a neutral baseline to minimize the bias introduced by long-term climatic features. Without this consideration, decadal variations in SSTs are prone to bias from climate change influences. Many studies tend to select a climatology period from the start of SST data availability but not to its end, leading to artificial trends in MHW_intensity_ for dates beyond the baseline period.

Therefore, based on the findings of this study, it can be concluded that MHWs in the Baltic Sea occur over a broader area each year with the same intensity. This highlights the importance of establishing a fixed baseline, emphasizing area as a crucial characteristic with a reasonable start and end date, and conducting a comprehensive analysis of a holistic MHW_index_ that incorporates all relevant characteristics to better understand MHWs in such basins. Furthermore, we investigated the association between sea surface heat flux components, as the indicators of internal drivers, averaged for each sub-basin in the Baltic Sea and MHW characteristics, with separate evaluations for cool and warm months. During cooler months, SSR is a major driver of MHW_intensity_ due to its direct contribution of heat to the sea surface. The role of STR during this period is relatively limited because atmospheric conditions decrease the effectiveness of heat loss through radiation. Consequently, more heat is retained at the sea surface, which supports the maintenance or intensification of MHWs, particularly in the southern subbasins where SSR input is significant, while there is no correlation between solar radiation and MHWs in the central and northern subbasins, since solar radiation generally decreases with increasing latitude. In contrast, during warmer months when daytime hours increase, SSR continues to influence MHW_intensity_, but the role of evaporative heat loss through surface SLHF becomes more significant as well. Increased convective processes during warmer months enhance cooling at the sea surface. However, this cooling effect is insufficient to fully counteract the warming effects driven by high SSR. Despite the substantial role of SLHF in cooling, the net impact of SSR remains dominant, leading to continued warming. These findings emphasize the critical role of solar radiation in shaping MHW characteristics throughout the year. The varying impacts of heat flux components—SSR, STR, and SLHF—highlight how seasonal atmospheric conditions modulate their effects. During cooler months, reduced atmospheric heat loss through STR results in greater heat retention, while in warmer months, the increased cooling from SLHF does not completely offset the warming effects of SSR. Thus, the interplay among these heat flux components and seasonal atmospheric conditions influences the intensity and spatial extent of MHWs in the Baltic Sea.

In addition, as a preliminary survey of the potential external drivers of MHWs in the Baltic Sea, the correlation analysis reveals seasonal variations in how global-scale climate oscillations impact MHW characteristics. Although the correlation coefficients are relatively modest, the results suggest that AO and NAO have a more pronounced influence on MHW characteristics during cooler months. Conversely, during warmer months, IOD and PDO appear to play a considerable role. These seasonal differences can be attributed to changes in oceanic and atmospheric conditions, which affect how heat is distributed and retained in the Baltic Sea. For cooler months, AO and NAO influence local weather patterns, which directly affect heat retention. In warmer months, the effects of IOD and PDO may be more indirect, mediated through broader atmospheric circulation patterns rather than direct local impacts. This variation highlights the complex interactions between climate oscillations and regional MHW characteristics, influenced by seasonal shifts in environmental conditions. Nevertheless, despite the lack of significant correlations between large-scale global climate oscillations and MHW characteristics, it has been demonstrated that the most influential external drivers in the Baltic Sea are those originating from the Northern Hemisphere. In contrast, climate oscillations from the Southern Hemisphere, such as ENSO and AAO, have an insufficient and negligible impact on MHW variability in the Baltic Sea. To better understand these potential external drivers of MHWs, this study focused on exploring correlations between MHW characteristics and several Northern Hemisphere teleconnection patterns that originate even closer to the Baltic Sea.

In this regard, the results illustrate that the more correlated teleconnection pattern with MHWs in the Baltic Sea is EA means the MHW characteristics are more influenced by the second prominent mode of low-frequency variability over the North Atlantic, which appears as a leading mode in all months^53,54^. Previously, the findings of Gröger et al. (2024) showed that the summer MHWs in the Baltic are due to local meteorological conditions caused by a dominant blocking over Scandinavia promoting anomalous strong shortwave downflux, calm winds, and low vertical mixing with colder sub-thermocline waters. And the winter MHWs are linked to the advection of warm and moist air originating from the North Atlantic^40^. On the other hand, the EA pattern is often interpreted as a “southward shifted” NAO pattern. According to the results of this study, it can be expected that while the Baltic MHWs are not perfectly connected to the NAO, the conditions for the occurrence of Baltic MHWs are more favorable when a southward shift occurs in the NAO. This southward shift aligns with the EA, indicating that during these periods, atmospheric and oceanic conditions conducive to MHW formation in the Baltic Sea are more likely to be present. Considering MHW_index_ in the warmer months, the second most significant pattern in northern hemisphere teleconnections correlating with MHWs in the Baltic Sea is SCAND, which corresponds to Scandinavian blocking. SCAND consists of a primary circulation center over Scandinavia, with weaker centers of opposite sign over western Europe and eastern Russia/western Mongolia. The positive phase of SCAND is associated with positive height anomalies, often reflecting major blocking anticyclones, over Scandinavia and northwestern Russia, while the negative phase is associated with negative height anomalies in these regions^55,56^. The present study also found that in the warmer months, the EA/WR accompanied by EP/NP has a negative correlation with the intensity and duration of MHWs in the Baltic Sea, with this negative correlation decreasing from the northern to the southern sub-basins. The EA/WR pattern, is one of the three prominent teleconnection patterns affecting Eurasia year-round. The positive phase is associated with positive height anomalies over Europe and northern China and negative height anomalies over the central North Atlantic and north of the Caspian Sea. Surface temperature anomalies during the positive phase reflect above-average temperatures in eastern Asia and below-average temperatures in western Russia which reaches to the Eastern and Northern Baltic Sea^57–59^. So, the positive phase of EA/WR create conditions less conducive to the formation and intensification of MHWs in the Baltic Sea, especially in the northern sub-basins. The patterns that can impact atmospheric circulation could lead to cooler temperatures and increased cloud cover or precipitation, reducing solar radiation and heat flux into the sea, thereby mitigating the intensity of MHWs in the Baltic Sea which is illustrated by the negative correlation coefficients in the present study.

To sum up, regarding internal and external drivers, this study sheds light on the variation of MHWs characteristics in the Baltic Sea, highlighting the distinct influences of both types of drivers across different seasons and sub-basins. The analysis reveals that in warmer months, MHWs are shaped by a complex interplay of internal and external drivers, with varying impacts observed across different sub-basins. Conversely, the drivers of MHWs during cooler months are still less well understood. However, this study offers important clues regarding the partial effects of both internal and external factors in cooler months for several subbasins. Therefore, to enhance understanding of MHWs in cooler months, future research can investigate additional oceanic and atmospheric variables, such as ice extent and other wintertime conditions specific to the Baltic Sea. This approach could provide clearer insights into the mechanisms driving MHWs during colder periods.

## Data and methods

### SST Data

This study utilizes a reprocessed SST data product from Copernicus Marine Service, with a product id of SST-BAL-SST-L4-REP-OBSERVATIONS-010-016, a multi-sensor, level 4 optimally interpolated product using infra-red satellite observations^60,61^. Covering a temporal extent of 42 years from 1982 to 2023, the dataset offers daily satellite reprocessed observations with a spatial resolution of 0.02° × 0.02°.

### MHW detection

Our approach for detecting MHWs from SST data is based on the method outlined by Hobday et al. (2016) which has been implemented in a MATLAB toolbox^17,46,62^. Our MHW detection is based on SST exceeding the 90th percentile of the baseline period for at least five consecutive days at each data grid. In addition, we hypothesized that the definition of a time-fixed baseline for detection of MHWs, as a consequence of climate change, could be biased by some global climate phenomena^63–65^. Previous research has highlighted the significant correlation between the atmospheric and oceanic conditions of the Baltic region and variations in two key climatic indicators: NAO and AMO^66–68^.

**Fig. 8:**
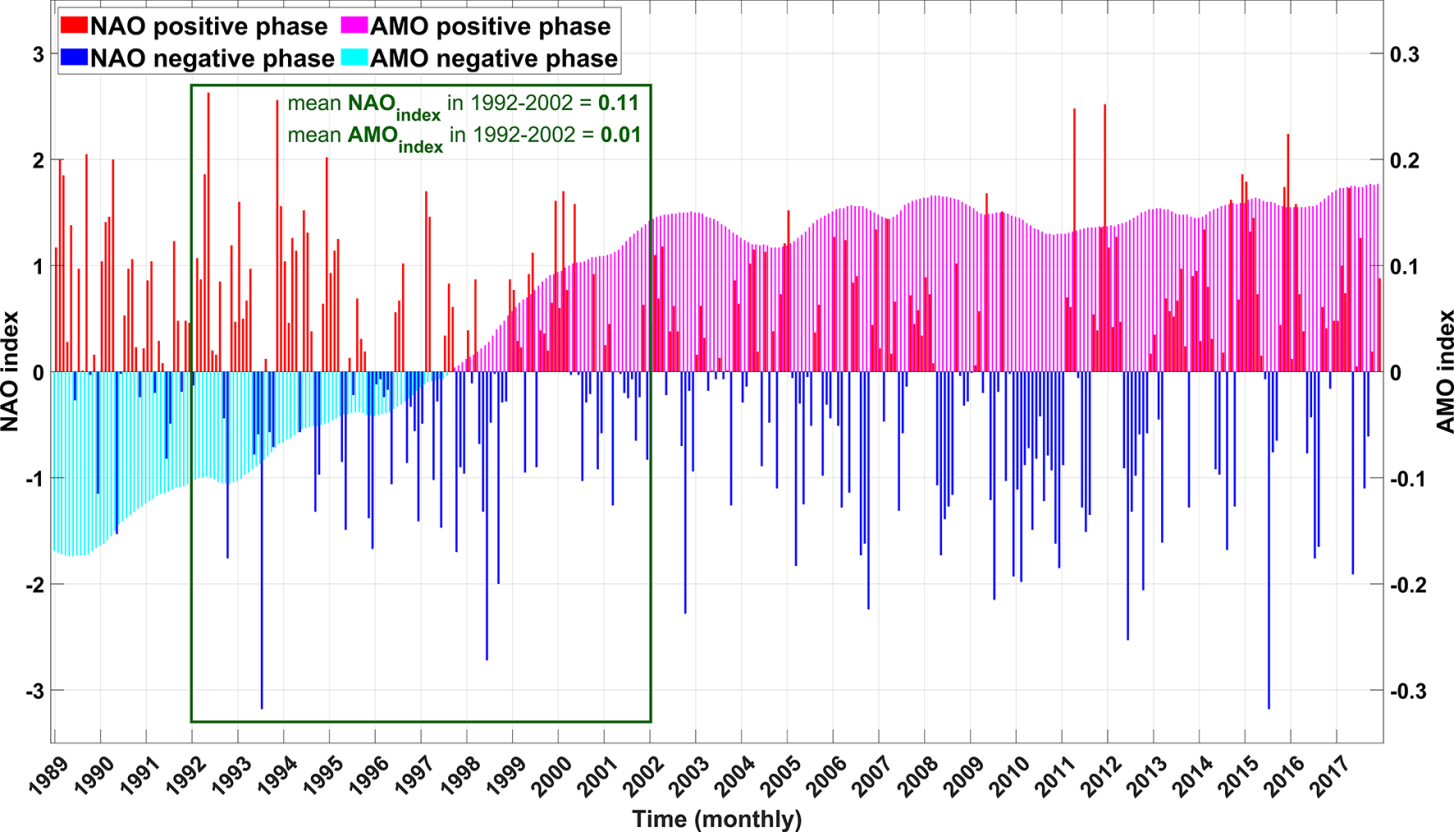
A neutral state period for the Baltic Sea in terms of NAO and AMO variability.

To mitigate the potential exaggeration of the impact of these indicators on defining a fixed-time baseline for MHW detection, their values were investigated for a long-term period in between the study period of this study. Figure 8 displays the monthly indices representing these climatic oscillations. The shorter period of analysis for these indices, compared to the SST data, is due to the availability of their monthly data from accessible public repositories. Nonetheless, these indices cover a significant portion of the study period and are considered adequate for the current study. On the other hand, it is suggested that the baseline period for conducting MHWs detection should include at least 10 continuous years^69^. Subsequently, employing a time window spanning a minimum duration of at least ten years, we observed a notable convergence wherein the average absolute values of both indices reached their nadir within the eleven-year interval from 1992 to 2002. This is how we established a neutral baseline from 1992 to 2002 to constrain the climatology in our Baltic Sea MHWs detection case study.

Using the provided data and criteria, MHWs in the Baltic Sea were detected for each sea grid point in the SST dataset over a 42-year period. The detection results produced daily maps of MHWs, which included values only if the SST at those grid points on that day exceeded the daily 90th percentile of the defined baseline period for at least five consecutive days.

### MHW characteristics

#### MHW_intensity_

The intensity of an MHW is measured by the degree to which the SST value in a grid point exceeds the 90th percentile threshold. In this study, the intensity of an MHW is measured by taking the average of the detected grid points for MHW in each daily map.

#### MHW_duration_

In this study, the duration of an MHW is the total number of consecutive days during which the SST at least at one grid point of the entire Baltic Sea or any subbasins, remains above the 90th percentile threshold.

Consequently, the characteristics of MHWs were determined to further evaluate them, starting with intensity and duration. However, it became evident during subsequent analysis that solely considering intensity and duration was inadequate due to the variability in detected MHW patterns. Some MHWs exhibited strong intensity despite affecting only a small number of grid points, while others impacted a large number of grid points with relatively low intensity. Therefore, the area of MHWs as a third characteristic became necessary to capture these varying patterns observed during our big data analysis.

#### MHW_area_

In the present study, the area covered by MHW, was evaluated by considering the integral of the area between detected grid points in each daily map of MHWs. Thus, each MHW was assessed based on three characteristics: intensity, duration, and area of inclusion during its realization period.

#### MHW_index_

Although, the previous three characteristics together facilitated the comparability of different events, they cannot individually provide an adequate basis for a comprehensive evaluation of different MHWs events in the present study. Then, to enhance the accuracy of our assessment and address the issue of comparability between different detected events, we devised a further characteristic as we’ve named MHW_Index_ (Eq. 1).

1 $$\:{{\text{M}\text{H}\text{W}}_{\text{i}\text{n}\text{d}\text{e}\text{x}}}_{T,S}={{\text{M}\text{H}\text{W}}_{\text{i}\text{n}\text{t}\text{e}\text{n}\text{s}\text{i}\text{t}\text{y}}}_{T,S}\times\:\frac{{{\text{M}\text{H}\text{W}}_{\text{d}\text{u}\text{r}\text{a}\text{t}\text{i}\text{o}\text{n}}}_{T,S}}{T}\:\times\:\frac{{{\text{M}\text{H}\text{W}}_{\text{a}\text{r}\text{e}\text{a}}}_{T,S}}{\text{S}}\:$$

For example, if we are going to investigate the MHW_index_, in one particular month (with daily length of “T”) in a particular basin with total area of “S” (in this study, S could be the area of the entire Baltic Sea or any of its subbasins), the $\:{{\text{M}\text{H}\text{W}}_{\text{d}\text{u}\text{r}\text{a}\text{t}\text{i}\text{o}\text{n}}}_{T,S}\:$is the sum of the days involving any detected MHW within T and S, $\:{{\text{M}\text{H}\text{W}}_{\text{i}\text{n}\text{t}\text{e}\text{n}\text{s}\text{i}\text{t}\text{y}}}_{T,S}\:$ and $\:{{\text{M}\text{H}\text{W}}_{\text{A}\text{r}\text{e}\text{a}}}_{T,S}\:$are the average of the only detected daily MHW_intensity_ and MHW_area_ in S and over $\:{{\text{M}\text{H}\text{W}}_{\text{d}\text{u}\text{r}\text{a}\text{t}\text{i}\text{o}\text{n}}}_{T,S}$, respectively.

### Exploration of potential drivers of MHWs

The study aimed to explore the potential drivers of MHWs in the Baltic Sea and its sub-basins, categorizing these drivers into external and internal factors. To investigate internal drivers, monthly mean maps of surface heat flux components — SSR, STR, SLHF, and SSHF — were extracted for the Baltic Sea and its subbasins in 1982–2023 from the global atmospheric reanalysis dataset ERA5^70^ and spatially averaged for each subbasin in the present study based on Fig. 1. The net heat flux was calculated by summing these components, taking into account their respective signs (with positive values indicating heat gained by the ocean).

Regarding external drivers, this study assumed that the signatures of these drivers are manifested in climatic oscillations. Therefore, six climatic oscillations, as global-scale potential influencing factors, including NAO, AO, IOD, ENSO, PDO, and AAO, were initially considered. In fact, for the selection of these climatic indices, it has been tried to consider representatives from each major oceanic area worldwide.

Additionally, to further evaluate the potential external drivers of MHWs in the Baltic, this study focused on northern hemisphere teleconnection patterns with less geographical distance from their originations to the Baltic Sea. Therefore, the relationships between EA, EA/WR, SCAND, POL, EP/NP, and PNA patterns were separately surveyed. Table 1 provides further details about the climate oscillations and patterns analyzed to find meaningful relationships with the characteristics of MHWs in the Baltic Sea and its sub-basins in the present study.

**Table 1:** Definitions of the climate patterns, including global-scaled climate oscillations and northern hemisphere teleconnections.

Abr.	Long name	Description
AAO	Antarctic Oscillation	The AAO index is constructed by projecting the (00Z) 700mb height anomalies poleward of 20°S onto the loading pattern of the AAO which is defined as the leading mode of Empirical Orthogonal Function (EOF) analysis of monthly mean 700 hPa height
AO	Arctic Oscillation	AO index is obtained by projecting the AO loading pattern to the anomaly 1000 millibar height field over 20°N-90°N latitude. The AO loading pattern has been chosen as the first mode of EOF analysis using monthly mean 1000 millibar height anomaly data over 20°N-90°N.
ENSO	El Niño-Southern Oscillation	ENSO index (Multivariate: MEI.v2) is the time series of the leading combined EOF of five different variables (sea level pressure (SLP), SST, zonal and meridional components of the surface wind, and outgoing longwave radiation (OLR)) over the tropical Pacific basin (30°S-30°N and 100°E-70°W).
IOD	Indian Ocean Dipole	IOD index is represented by anomalous SST gradient between the western equatorial Indian Ocean (50E-70E and 10 S–10 N) and the south eastern equatorial Indian Ocean (90E-110E and 10 S–0 N).
NAO	North Atlantic Oscillation	NAO index is based on the surface sea-level pressure difference between the Subtropical (Azores) High and the Subpolar Low. The NAO index is obtained by projecting the NAO loading pattern to the daily anomaly 500 millibar height field over 0–90°N. The NAO loading pattern has been chosen as the first mode of a Rotated EOF analysis using monthly mean 500 millibar height anomaly data from 1950 to 2000 over 0–90°N latitude.
PDO	Pacific Decadal Oscillation	PDO index is based on NOAA’s extended reconstruction of SSTs (ERSST Version 5). When SSTs are anomalously cool in the interior North Pacific and warm along the Pacific Coast, and when sea level pressures are below average over the North Pacific, the PDO has a positive value. When the climate anomaly patterns are reversed, with warm SST anomalies in the interior and cool SST anomalies along the North American coast, or above average sea level pressures over the North Pacific, the PDO has a negative value
EA	East Atlantic pattern	The EA pattern is the second prominent mode of low-frequency variability over the North Atlantic. It resembles the North Atlantic Oscillation (NAO) but is characterized by a southward shift. It consists of a north-south dipole of anomaly centers spanning the North Atlantic from east to west. The lower-latitude center contains a strong subtropical link, making the EA pattern distinct from the NAO.
EA/WR	East Atlantic/Western Russia pattern	The EA/WR pattern is one of three prominent teleconnection patterns affecting Eurasia throughout the year. It consists of four main anomaly centers, with the positive phase associated with positive height anomalies over Europe and northern China, and negative anomalies over the central North Atlantic and north of the Caspian Sea.
SCAND	Scandinavia pattern	SCAND consists of a primary circulation center over Scandinavia, with weaker centers over western Europe and eastern Russia/western Mongolia. The positive phase is associated with positive height anomalies over Scandinavia and western Russia, during the positive phase of SCAND, there is an occurrence of a blocking high-pressure system over Scandinavia, often referred to as “Scandinavian Blocking”, while the negative phase is associated with negative height anomalies in these regions.
POL	Polar/Eurasia pattern	POL appears in all seasons and consists of negative height anomalies over the polar region and positive anomalies over northern China and Mongolia. It is associated with fluctuations in the strength of the circumpolar circulation and is mainly associated with temperature and precipitation anomalies in eastern Siberia and China.
EP/NP	East Pacific-North Pacific pattern	EP/NP is a Spring-Summer-Fall pattern with three main anomaly centers. The positive phase features positive height anomalies over Alaska/Western Canada and negative anomalies over the central and eastern North Pacific. It is associated with surface temperature and precipitation anomalies over the eastern North Pacific and western North America.
PNA	Pacific/North American pattern	PNA is one of the most prominent modes of low-frequency variability in the Northern Hemisphere extratropic. It features above-average heights in the vicinity of Hawaii and over the intermountain region of North America in its positive phase. It is associated with fluctuations in the strength and location of the East Asian jet stream and surface temperature and precipitation anomalies over North America.

After preparing monthly time series data for the potential internal and external drivers, as well as the MHW characteristics, the datasets were divided into two sets based on time-based segmentation. This division was primarily motivated by a recent study that found the drivers of MHW events differ between the cool and warm months of the year^40^. Additionally, Fig. 3 in the current study illustrates variations in seasonal spatial patterns across four seasons. However, a preliminary overview suggests that the patterns in the first and fourth seasons (Fig. 3a and d) are similar, as are the patterns in the second and third seasons (Fig. 3b and c). To streamline the results and avoid redundancy, the study grouped the seasons into two periods: (1) December to May (cool months) and (2) June to November (warm months). Separate time series were then created for each period, based on the average of the cool and warm months in each year, respectively, for each of the internal and external drivers, as well as the MHW characteristics.

The study then analyzed the correlation between these seasonal mean time series over the 42-year study period. This analysis was conducted first by examining the potential internal drivers (i.e., heat flux components) in relation to MHW characteristics, and then by analyzing the potential external drivers (i.e., climate oscillations and teleconnection indices) in relation to MHW characteristics, separately. The correlation analysis was based on Pearson correlation coefficients (Eq. 2) and P-values (Eq. 3)^71–73^.

2 $$\:{\text{C}\text{o}\text{r}\text{r}\text{e}\text{l}\text{a}\text{t}\text{i}\text{o}\text{n}\:\text{c}\text{o}\text{e}\text{f}\text{f}\text{i}\text{c}\text{i}\text{e}\text{n}\text{t}}_{{MHW}_{CHAR},{Driver}_{x}}:\text{r}=\frac{1}{N-1}\sum\:_{\varvec{i}=1}^{\varvec{N}}\left(\frac{{\varvec{M}\varvec{H}\varvec{W}}_{{\varvec{C}\varvec{H}\varvec{A}\varvec{R}}_{\varvec{i}}}-{\varvec{\mu\:}}_{{\varvec{M}\varvec{H}\varvec{W}}_{{\varvec{C}\varvec{H}\varvec{A}\varvec{R}}_{\varvec{i}}}}}{{\varvec{\sigma\:}}_{{\varvec{M}\varvec{H}\varvec{W}}_{{\varvec{C}\varvec{H}\varvec{A}\varvec{R}}_{\varvec{i}}}}}\right)\left(\frac{{\varvec{D}\varvec{r}\varvec{i}\varvec{v}\varvec{e}\varvec{r}}_{{\varvec{x}}_{\varvec{i}}}-{\varvec{\mu\:}}_{{\varvec{D}\varvec{r}\varvec{i}\varvec{v}\varvec{e}\varvec{r}}_{{\varvec{x}}_{\varvec{i}}}}}{{\varvec{\sigma\:}}_{{\varvec{D}\varvec{r}\varvec{i}\varvec{v}\varvec{e}\varvec{r}}_{{\varvec{x}}_{\varvec{i}}}}}\right)\:$$

Where *N* is the number of time steps. $\:\varvec{\mu\:}$ and $\:\varvec{\sigma\:}$ are the operators for the mean and standard deviation respectively. *CHAR* includes intensity, duration, area and index and x represents each potential MHW driver considered in the present study. Thus, the correlation was calculated between each specific driver and each of the MHW characteristics (CHAR). Then, Correlation coefficients were considered statistically insignificant if the P-value exceeded 0.05, in which case they were excluded from the resulting plots (Figs. 5 and 6, and 7).

3 $$\:{\text{P}-\text{v}\text{a}\text{l}\text{u}\text{e}}_{{MHW}_{CHAR},{Driver}_{x}}:\text{p}=2\times\:tcdf\left(-\left|t\right|,df\right)\:$$

Where ∣t∣ is the absolute value of the t-statistic (Eq. 4). The ‘*tcdf’* operator denotes the cumulative distribution function of the t-distribution, and *df* represents the degrees of freedom, which is equal to N − 2.

4 $$\:\text{t}=\frac{r\sqrt{N-2}}{\sqrt{1-{r}^{2}}}\:$$

## Data Availability

The datasets utilized in this study are publicly available and can be accessed from the following sources: 1. The SST data which has been analyzed in this study are available at Copernicus Marine Service under the name of Baltic Sea - Sea Surface Temperature Reprocessed: (https://data.marine.copernicus.eu/product/SST_BAL_SST_L4_REP_OBSERVATIONS_010_016/) There is also a Quality Information Document including the validation information. 2. ERA5 heat flux components data are available at Climate Data Store, Copernicus Climate Change Service (https://cds.climate.copernicus.eu/)0. 3. The large-scale climate oscillations monthly timeseries data including NAO, AO, IOD, ENSO, PDO, and AAO are available at: https://psl.noaa.gov/. 4. Monthly timeseries data for AMO available at: https://climatedataguide.ucar.edu/. 5. Monthly timeseries data for the northern hemisphere teleconnection patterns available at: https://www.cpc.ncep.noaa.gov/data/teledoc/telecontents.shtml. All other datasets presented in this study are outcomes derived from the procedures outlined in Sect. 4, and they can be replicated using the aforementioned data. Nevertheless, all datasets resulted in this study are available from the corresponding author upon reasonable request.

## References

[1] Pearce, A. F. et al. *The Marine heat wave off Western Australia during the Summer of 2010/11* (Western Australian Fisheries and Marine Research Laboratories, 2011).

[2] Wild, S. et al. Long-term decline in survival and reproduction of dolphins following a marine heatwave. *Curr. Biol.* **29**, R239–R240 (2019). 30939303 10.1016/j.cub.2019.02.047

[3] Ainsworth, T. D., Hurd, C. L., Gates, R. D. & Boyd, P. W. How do we overcome abrupt degradation of marine ecosystems and meet the challenge of heat waves and climate extremes? *Glob Chang. Biol.* **26**, 343–354 (2020). 31873988 10.1111/gcb.14901

[4] Samhouri, J. et al. (ed, F.) Marine heatwave challenges solutions to human–wildlife conflict. *Proc. R Soc. B* **288** 20211607 (2021). 34847764 10.1098/rspb.2021.1607 PMC8634617

[5] Stipcich, P. et al. Effects of current and future summer marine heat waves on Posidonia oceanica: plant origin matters? *Front. Clim.* **4**, 844831 (2022).

[6] Burger, F. A., Terhaar, J. & Frölicher, T. L. Compound marine heatwaves and ocean acidity extremes. *Nat. Commun.* **13**, 4722 (2022). 35973999 10.1038/s41467-022-32120-7 PMC9381716

[7] Perkins-Kirkpatrick, S. E. et al. Natural hazards in Australia: heatwaves. *Clim. Change*. **139**, 101–114 (2016).

[8] Oliver, E. C. J. et al. Longer and more frequent marine heatwaves over the past century. *Nat. Commun.* **9**, 1–12 (2018). 29636482 10.1038/s41467-018-03732-9 PMC5893591

[9] Dzwonkowski, B. et al. Compounding impact of severe weather events fuels marine heatwave in the coastal ocean. Nat Commun 11: 4623. at (2020). 10.1038/s41467-020-18339-2 PMC7508827 32963230

[10] Elzahaby, Y., Schaeffer, A., Roughan, M. & Delaux, S. Oceanic circulation drives the deepest and longest marine heatwaves in the East Australian Current system. *Geophys. Res. Lett.* 48, eGL094785 (2021). (2021).

[11] Johnson, G. C. & Lyman, J. M. Warming trends increasingly dominate global ocean. *Nat. Clim. Chang.* **10**, 757–761 (2020).

[12] Yamaguchi, R. & Suga, T. Trend and variability in global upper-ocean stratification since the 1960s. *J. Geophys. Res. Ocean.* **124**, 8933–8948 (2019).

[13] Ruela, R., Sousa, M. C., DeCastro, M. & Dias, J. M. Global and regional evolution of sea surface temperature under climate change. *Glob Planet. Change*. **190**, 103190 (2020).

[14] Frölicher, T. L. & Fischer, E. M. Gruber, N. Marine heatwaves under global warming. *Nature*. **560**, 360–364 (2018). 30111788 10.1038/s41586-018-0383-9

[15] Smale, D. A., Wernberg, T. & Vanderklift, M. A. Regional-scale variability in the response of benthic macroinvertebrate assemblages to a marine heatwave. *Mar. Ecol. Prog Ser.* **568**, 17–30 (2017).

[16] Sen Gupta, A. et al. Drivers and impacts of the most extreme marine heatwave events. *Sci. Rep.* **10**, 19359 (2020). 33168858 10.1038/s41598-020-75445-3 PMC7653907

[17] Hobday, A. J. et al. A hierarchical approach to defining marine heatwaves. *Prog Oceanogr.* **141**, 227–238 (2016).

[18] Hobday, A. J. et al. Categorizing and naming marine heatwaves. *Oceanography*. **31**, 162–173 (2018).

[19] Ibrahim, O., Mohamed, B. & Nagy, H. Spatial variability and trends of marine heat waves in the eastern mediterranean sea over 39 years. *J. Mar. Sci. Eng.* **9**, 643 (2021).

[20] Mohamed, B., Ibrahim, O. & Nagy, H. Sea Surface temperature variability and marine heatwaves in the black sea. *Remote Sens.* **14**, 2383 (2022).

[21] Kashkooli, O. B., Karimian, S. & Modarres, R. Spatiotemporal variability of the Persian Gulf and Oman Sea Marine heatwaves during 1982–2020. *Mar. Pollut Bull.* **184**, 114174 (2022). 36194961 10.1016/j.marpolbul.2022.114174

[22] Kumar, S. et al. Analysis of marine heatwaves over the Bay of Bengal during 1982–2021. *Sci. Rep.* **13**, 14235 (2023). 37648697 10.1038/s41598-023-39884-y PMC10468509

[23] Wang, H., Lu, Y., Zhai, L., Chen, X. & Liu, S. Variations of surface marine heatwaves in the Northwest Pacific during 1993–2019. *Front. Mar. Sci.* **11**, 1323702 (2024).

[24] Marbà, N., Jordà, G., Agustí, S., Girard, C. & Duarte, C. M. Footprints of climate change on Mediterranean Sea Biota. *Front. Mar. Sci.* **2**, 56 (2015).

[25] Darmaraki, S., Somot, S., Sevault, F. & Nabat, P. Past variability of Mediterranean Sea Marine heatwaves. *Geophys. Res. Lett.* **46**, 9813–9823 (2019).

[26] Oliver, E. C. J. et al. Marine heatwaves. *Ann. Rev. Mar. Sci.* **13**, 313–342 (2021). 32976730 10.1146/annurev-marine-032720-095144

[27] Rosselló, P., Pascual, A. & Combes, V. Assessing marine heat waves in the Mediterranean Sea: a comparison of fixed and moving baseline methods. *Front. Mar. Sci.* **10**, 1168368 (2023).

[28] Moron, V., Vautard, R. & Ghil, M. Trends, interdecadal and interannual oscillations in global sea-surface temperatures. *Clim. Dyn.* **14**, 545–569 (1998).

[29] Steinman, B. A., Mann, M. E. & Miller, S. K. Atlantic and Pacific multidecadal oscillations and Northern Hemisphere temperatures. *Sci. (80-)*. **347**, 988–991 (2015). 10.1126/science.1257856 25722410

[30] Schrum, C. & Backhaus, J. O. Sensitivity of atmosphere–ocean heat exchange and heat content in the North Sea and the Baltic Sea. *Tellus A*. **51**, 526–549 (1999).

[31] Döscher, R. & Meier, H. E. M. Simulated sea surface temperature and heat fluxes in different climates of the Baltic Sea. *AMBIO J. Hum. Environ.* **33**, 242–248 (2004). 15264603

[32] Voss, R. et al. Ecological-economic sustainability of the Baltic Cod fisheries under ocean warming and acidification. *J. Environ. Manage.* **238**, 110–118 (2019). 30849595 10.1016/j.jenvman.2019.02.105

[33] Reusch, T. B. H. et al. The Baltic Sea as a time machine for the future coastal ocean. *Sci. Adv.* **4**, eaar8195 (2018). 29750199 10.1126/sciadv.aar8195 PMC5942908

[34] Lehmann, A. et al. Salinity dynamics of the Baltic Sea. *Earth Syst. Dyn.* **13**, 373–392 (2022).

[35] Raudsepp, U., Maljutenko, I., Barzandeh, A., Uiboupin, R. & Lagemaa, P. Baltic Sea freshwater content. *State Planet.* **1**, 1–14 (2023).

[36] Placke, M. et al. Long-term mean circulation of the Baltic Sea as represented by various ocean circulation models. *Front. Mar. Sci.* **5**, 287 (2018).

[37] Tanaka, K. R. & Van Houtan, K. The recent normalization of historical marine heat extremes. *PloS Clim.* **1**, e0000007 (2022).

[38] Meier, H. E. M. et al. Climate change in the Baltic Sea region: a summary. *Earth Syst. Dyn.* **13**, 457–593 (2022).

[39] Rutgersson, A. et al. Natural hazards and extreme events in the Baltic Sea region. *Earth Syst. Dyn.* **13**, 251–301 (2022).

[40] Gröger, M., Dutheil, C., Börgel, F. & Meier, M. H. E. drivers of marine heatwaves in a stratified marginal sea. *Clim. Dyn.* 1–13 (2024).

[41] Travkin, V. S., Tikhonova, N. A. & Zakharchuk, E. A. Characteristics of Marine heatwaves of the Baltic Sea for 1993 – 2022 and their driving factors. *Pure Appl. Geophys.* 1–15 (2024).

[42] Dabulevičienė, T. & Servaitė, I. Characteristics of Marine heatwaves in the Southeastern Baltic Sea Based on long-term in situ and Satellite observations. *J. Mar. Sci. Eng.* **12**, 1109 (2024).

[43] Pinto, G. et al. Longer and more frequent marine heatwaves in the Western Baltic Sea. https://www.researchsquare.com/article/rs-3910435/v1 (2024). 10.21203/rs.3.rs-3910435/v1

[44] Safonova, K., Meier, H. E. M. & Gröger, M. Summer heatwaves on the Baltic Sea seabed contribute to oxygen deficiency in shallow areas. *Commun. Earth Environ.* **5**, 106 (2024).

[45] Book, I. I. G. C. & Contributors, O. D. GEBCO_2019 Grid.

[46] Inc., T. M. MATLAB version: 9.13.0 (R at (2022). https://www.mathworks.com (2022).

[47] Humborg, C. et al. High emissions of carbon dioxide and methane from the coastal Baltic Sea at the end of a summer heat wave. *Front. Mar. Sci.* **6**, 493 (2019).

[48] Suursaar, Ü. Combined impact of summer heat waves and coastal upwelling in the Baltic Sea. *Oceanologia*. **62**, 511–524 (2020).

[49] Cahill, B., Chrysagi, E., Vortmeyer-Kley, R. & Gräwe, U. Deconstructing co-occurring marine heatwave and phytoplankton bloom events in the Arkona Sea in 2018. *Front. Mar. Sci.* **11**, 1323271 10.3389/fmars.2024.1323271 (2024).

[50] Chiswell, S. M. Global trends in marine heatwaves and cold spells: the impacts of fixed versus changing baselines. *J. Geophys. Res. Ocean.* **127**, e2022JC018757 (2022).

[51] Li, X., Wu, R., Dai, P., Cai, R. & Tan, H. Diverse marine heatwave intensity trends in the marginal seas of China. *Theor. Appl. Climatol* 1–14 (2024).

[52] Mohamed, B., Nilsen, F. & Skogseth, R. Marine heatwaves characteristics in the Barents Sea based on high resolution satellite data (1982–2020). *Front. Mar. Sci.* **9**, 821646 (2022).

[53] Moore, G. W. K. & Renfrew, I. A. Cold European winters: interplay between the NAO and the East Atlantic mode. *Atmos. Sci. Lett.* **13**, 1–8 (2012).

[54] Simpson, I., Hanna, E., Baker, L., Sun, Y. & Wei, H. North Atlantic atmospheric circulation indices: links with summer and winter temperature and precipitation in north-west Europe, including persistence and variability. *Int. J. Climatol* (2024).

[55] Liu, Y., Wang, L., Zhou, W. & Chen, W. Three eurasian teleconnection patterns: spatial structures, temporal variability, and associated winter climate anomalies. *Clim. Dyn.* **42**, 2817–2839 (2014).

[56] Pang, B., Scaife, A. A., Lu, R., Ren, R. & Zhao, X. Interdecadal variations of the scandinavian pattern. *J. Clim.* **36**, 3275–3288 (2023).

[57] Krichak, S. O. & Alpert, P. Decadal trends in the east Atlantic–West Russia pattern and Mediterranean precipitation. *Int. J. Climatol J. R Meteorol. Soc.* **25**, 183–192 (2005).

[58] Lim, Y. K. The East Atlantic/West Russia (EA/WR) teleconnection in the North Atlantic: climate impact and relation to Rossby wave propagation. *Clim. Dyn.* **44**, 3211–3222 (2015).

[59] Sukhonos, O. & Vyshkvarkova, E. Connection of compound extremes of Air Temperature and Precipitation with Atmospheric circulation patterns in Eastern Europe. *Climate*. **11**, 98 (2023).

[60] Høyer, J. L. & She, J. Optimal interpolation of sea surface temperature for the North Sea and Baltic Sea. *J. Mar. Syst.* **65**, 176–189 (2007).

[61] Høyer, J. L. & Karagali, I. Sea surface temperature climate data record for the North Sea and Baltic Sea. *J. Clim.* **29**, 2529–2541 (2016).

[62] Zhao, Z. & Marin, M. A MATLAB toolbox to detect and analyze marine heatwaves. *J. Open. Source Softw.* **4**, 1124 (2019).

[63] Salinger, J. et al. Decadal-scale forecasting of climate drivers for marine applications. *Adv. Mar. Biol.* **74**, 1–68 (2016). 27573049 10.1016/bs.amb.2016.04.002

[64] Thomsen, M. et al. Drivers and impacts of the most extreme marine heatwaves events. *Sci. Rep.* **10**, 19359 (2020). 33168858 10.1038/s41598-020-75445-3 PMC7653907

[65] Dong, L. et al. Roles of external forcing and internal variability in global marine heatwaves change during 1982–2021. *Geophys. Res. Lett.* 51, eGL107557 (2024). (2023).

[66] Börgel, F., Frauen, C., Neumann, T., Schimanke, S. & Meier, H. E. M. Impact of the Atlantic multidecadal oscillation on Baltic Sea variability. *Geophys. Res. Lett.* **45**, 9880–9888 (2018).

[67] Kniebusch, M., Meier, H. E. M., Neumann, T. & Börgel, F. Temperature variability of the Baltic Sea since 1850 and attribution to atmospheric forcing variables. *J. Geophys. Res. Ocean.* **124**, 4168–4187 (2019).

[68] Barroso, A. et al. Observed spatiotemporal variability in the annual sea level cycle along the global coast. *J. Geophys. Res. Ocean.* **129**, e2023JC020300 (2024).

[69] Spillman, C. M., Alves, O. & Hudson, D. A. Predicting thermal stress for coral bleaching in the great barrier reef using a coupled ocean–atmosphere seasonal forecast model. *Int. J. Climatol*. **33**, 1001–1014 (2013).

[70] Hersbach, H. et al. The ERA5 global reanalysis. *Q. J. R Meteorol. Soc.* **146**, 1999–2049 (2020).

[71] Kendall, M. G. The advanced theory of statistics. (1943).

[72] Fisher, R. A. Statistical methods for research workers. in Breakthroughs in Statistics: Methodology and Distribution 66–70 (Springer, (1970).

[73] Teukolsky, S. A., Flannery, B. P., Press, W. H. & Vetterling, W. Numerical recipes in C. *SMR*. **693**, 59–70 (1992).

