# Author Correction: Variability of marine heatwaves’ characteristics and assessment of their potential drivers in the Baltic Sea over the last 42 years

**DOI:** 10.1038/s41598-025-88630-z

**Published:** 2025-02-05

**Authors:** Behzad Bashiri, Amirhossein Barzandeh, Aarne Männik, Urmas Raudsepp

**Affiliations:** Department of Marine Systems, Tallinn University of Technology, 12618 Tallinn, Estonia

Correction to: *Scientific Reports* 10.1038/s41598-024-74173-2, published online 28 September 2024

In the original version of this Article, the authors omitted the below Reference, which is listed below as Reference 49.

49. **Cahill B, Chrysagi E, Vortmeyer-Kley R, and Gräwe U (2024).** *Deconstructing co-occurring marine heatwave and phytoplankton bloom events in the Arkona Sea in 2018.* Front. Mar. Sci. 11:1323271. doi: 10.3389/fmars.2024.1323271.

As a result, in the Introduction,

“Cahill et al. (2024) examined the interplay between MHWs and phytoplankton blooms in the Arkona Sea during 2018, using satellite data and modeling.”

now reads:

“Cahill et al. (2024) examined the interplay between MHWs and phytoplankton blooms in the Arkona Sea during 2018, using satellite data and modeling^49^.”

As a result of the changes, the References have been renumbered.

The original Article has been corrected.

